# Osteopontin Levels in Patients with Squamous Metastatic Head and Neck Cancer

**DOI:** 10.3390/medicina57020185

**Published:** 2021-02-21

**Authors:** Josip Maleš, Hrvoje Mihalj, Anamarija Šestak, Kristina Kralik, Martina Smolić

**Affiliations:** 1 Department of Otorhinolaryngology and Maxillofacial Surgery, Medical Faculty, University of Osijek, J. Huttlera 4, 31000 Osijek, Croatia; josipimales@gmail.com (J.M.); hrvoje.mihalj@gmail.com (H.M.); 2 Department of Otorhinolaryngology and Head and Neck Surgery, University Clinical Hospital Centre Osijek, J. Huttlera 4, 31000 Osijek, Croatia; annamarijasestak@gmail.com; 3 Faculty of Medicine Osijek, University of Osijek, J. Huttlera 4, 31000 Osijek, Croatia; kristina.kralik@gmail.com; 4 Department of Pharmacology, Faculty of Medicine, University of Osijek, J. Huttlera 4, 31000 Osijek, Croatia; 5 Department of Pharmacology and Biochemistry, Faculty of Dental Medicine and Health Osijek, University of Osijek, Crkvena ul. 1, 31000 Osijek, Croatia

**Keywords:** squamous cancer, osteopontin, neck metastases

## Abstract

*Background and Objectives*: Increased osteopontin (OPN) concentrations in the plasma of patients with head and neck squamous cancer (HNSCC) have diagnostic significance, and it can indicate more aggressive biological behavior of cancer. The aim of this study was to determine OPN levels in patients with HNSCC of different primary locations and to assess its prognostic significance in metastasis development. *Materials and Methods*: This cohort study included 45 patients (41 male and 4 female patients) with HNSCC with different primary localization of head and neck. All patients underwent surgery—neck dissection. All patients were categorized according to the histological findings of the resected material and tumor–node–metastasis (TNM) classification system. After surgery, N categories were determined on the basis of histological features of resected material. *Results*: The histological findings of our patients showed: N0 in 11 patients, N1 in 8 patients, N2a in 4 patients, N2b in 14 patients and N2c in 8 patients. Plasma OPN values in all study participants ranged from 2.24 to 109.10 ng/mL. OPN levels in plasma of patients with negative nodes compared to the group of patients with positive nodes in the neck differed significantly (16.89 ng/mL to 34.08 ng/mL, respectively; *p* = 0.03). There were significantly lower OPN plasma levels in the group of subjects with histologically positive one lymph node in the neck (N1) compared to the group of patients with N2b histologically positive findings of resected neck material (10.4 ng/mL to 43.9 ng/mL, respectively; *p* = 0.02). *Conclusions*: The results have shown that growing N degrees of positive neck nodes classification were accompanied by growing values of plasma osteopontin. Osteopontin might be important for the development of neck metastases.

## 1. Introduction

Osteopontin (OPN) is a glycoprotein synthesized by different cell types, including epithelial cells. Acting as a cytokine and as an extracellular matrix protein, OPN binds to receptors on the cell surface and thereby triggers the transmission of signals that regulate numerous physiological and pathological processes [1]. OPN can be detected in blood, and urine and it is known to be involved in cancer biology by regulating processes such as cell proliferation angiogenesis, invasion, and metastasis [2,3]. It is expressed in various cancer types like lung cancer, gastric cancer, breast cancer, and head and neck squamous cell carcinoma (HNSCC) [4,5,6,7]. Osteopontin is also found in mineralized tissues such as bone. In vitro studies show that OPN is firmly adsorbed to hydroxyapatite, an excellent scaffold for osteogenic proliferation and differentiation of osteoprogenitor cells, and acts as a crystal growth inhibitor. This is important in oral and maxillofacial surgery because hydroxyapatite ceramic-based materials are used for bone replacement [8].

Increased OPN concentrations in the plasma of patients with HNSCC have diagnostic significance, and it is used as a biomarker [9,10]. Overexpression of OPN can indicate more aggressive biological behavior of cancer and can be an important factor for survival [11]. In addition, it is found that positive OPN expression was correlated with clinical stage and cervical lymphatic metastasis in laryngeal squamous cell carcinoma [2,11]. The knowledge about the role of OPN in the biology of tumor growth is of great importance and can help in the timely identification of patients who will have a rapid disease progression and in the planning of appropriate therapy.

The current hypothesis about the origin of malignant tumors implies changes in the normal mechanism of cell proliferation and differentiation, and lack of apoptosis (cell death). These growth control changes result from mutations in specific genes in the process of which proto-oncogenes are activated and/or tumor suppressor genes are inactivated [12]. Changes in the phenotype enable cancer cells to have a growth advantage, free themselves from normal growth control, and enable growth defects in the final differentiation of cells [12]. Furthermore, biological aggressiveness of head and neck tumors, including cancer metastases, largely depends on the tumor hypoxia [13]. Increased OPN levels correlate with tumor hypoxia and are associated with worse overall survival in patients with HNSCC [14,15]. Additionally, tumor hypoxia is the main resistance factor to radiation treatment, and high OPN levels have a role in resistance to chemotherapy [14,16,17].

The aim of the study was to determine the plasma levels of osteopontin in patients with head and neck cancer prior to surgery, and to determine the possible correlation between osteopontin levels and the metastasis status in the neck lymph nodes of HCNSCC with different primary location. The secondary aim of the study was to assess the potential application of OPN as a molecular biomarker for prognosis and treatment of HNSCC with different primary localization.

## 2. Materials and Methods

### 2.1. Study Design

Between 2015 and 2019, 45 consecutively selected patients with HNSCC with different primary localization of head and neck were enrolled in the cohort study. All patients were treated at the University Hospital Center Osijek. Inclusion criteria were newly discovered HNSCC confirmed by the previous biopsy. Patients who did not undergo surgery, underwent certain oncology treatment before surgery or did not agree to sign informed consent to participate in the study were excluded from the study.

All patients underwent surgery—classic, modified, or selective dissection of one or both sides of the neck (or extended dissection), and all groups of lymph nodes from the resected material were identified and histologically categorized based on the criterion of presence or absence of lymph node metastasis, number and size of the affected lymph nodes, presence or absence of lymph node capsule infiltration. All patients were categorized according to the histological findings of the resected material and tumor–node–metastasis (TNM) classification system which was developed at the Memorial Sloan-Kettering Cancer Center) classification. 

Plasma samples were taken from all patients before surgical treatment to determine the OPN levels. With patient consent, a 5 mL blood sample was obtained by venipuncture into a vacutainer containing EDTA. The samples were centrifuged, and the separated plasma samples were removed and stored at −80 °C until they were assayed. OPN levels were determined in the laboratory of the University Hospital Center in Osijek by ELISA Quantikine HUMAN osteopontin assay as suggested by the manufacturer (R&D Systems, Thermo Fisher Scientific, Leicestershire, UK). 

After the plasma values of osteopontin were assessed, patients underwent neck dissection surgery. Subsequently, they were divided into two groups: a group of patients with negative pathological findings in resected material and a group of patients with positive histological findings of resected material.

### 2.2. Statistical Analysis

Categorical variables were described with percentages and numeric variables with means and interquartile range. The normality of the distribution of numeric variables was tested with the Kolmogorov–Smirnov test. We performed a two-step cluster analysis in the group of patients with histologically positive findings of resected neck material according to the histological classification of lymph nodes and metastases in the neck and plasma osteopontin levels. The difference between the two independent groups was tested with the Mann–Whitney U test. The difference between three and more groups was tested by the Kruskal–Wallis test. All *p* values were two-sided. The level of significance was set at alpha of 0.05. The statistical analysis was performed using MedCalc Statistical Software version 18 (MedCalc Software bvba, Ostend, Belgium; http://www.medcalc.org; accessed on 20 February 2021). 

## 3. Results

The study included 45 patients with HNSCC (41 male patients and four female patients). The mean age of the patients was 61 years (interquartile range IQR 41–74 years).

Primary head and neck carcinoma were localized in the larynx in 15 out of 45 study participants and in the tongue in 8 out of 45 study participants, whereas other localizations were present from 1 to 5 out of 45 study participants, respectively, as shown in Table 1.

Based on the extent of the primary tumor, participants were classified as illustrated in Table 2.

Plasma osteopontin values in all study participants ranged from 2.24 to 109.10 ng/mL (data not shown). 

Interestingly, OPN levels in the plasma of patients with histologically negative findings compared to a group with histologically positive neck nodes differed significantly (16.89 ng/mL vs. 34.08 ng/mL; *p* = 0.03) as shown in Table 3.

According to histological findings, medians and interquartile ranges of OPN values in groups of patients with histologically positive lymph nodes in the resected material showed a tendency to a positive association with the N stage of tumors. However, the highest OPN levels were determined in the group of patients with histologically confirmed N2b stage without statistical significance between the groups, as shown in Table 4.

According to data on plasma osteopontin levels and the histological classification of lymph nodes, for further interpretation of findings in the group of patients with histologically positive findings of resected neck material, the classification into clusters was made using two-step cluster analysis.

According to the cluster classification among the N groups of the classification based on histopathologic findings of resected material, two groups were homogeneous: all eight members of cluster one are patients with N1 neck, and in cluster three, there were 14 patients with N2b (metastasis in multiple ipsilateral lymph nodes, no larger than 6 cm). Cluster 2 consists of patients with N2a (four patients) and N2c (eight patients) neck status, as shown in Table 5. 

As these are (N1 and N2b) relatively large groups for the comparison of OPN plasma levels in patients with metastasis in the resected material, we conducted the test for OPN concentrations in these two segments of the patient population with neck metastasis.

There were significantly lower OPN plasma levels in the group of subjects with histologically positive one lymph node in the neck (N1) compared to a group of patients with N2b histologically positive findings of resected neck material (Mann Whitney U test, *p* = 0.02), while there were no significant differences between the other groups (Table 6).

## 4. Discussion

In recent years, new avenues of research have reinforced the understanding of the metastasis process. Furthermore, several models have been restored, and for a number of research directions the idea of their role in the metastasis process has been modified. 

For example, epithelial–mesenchymal transition enables tumor cells to have characteristics similar to embryonic cells, increasing their dispersal, survival, and growth ability in distant places, and this renews the idea that tumors are in many ways similar to embryonic processes. Furthermore, as the micro-surroundings of a tumor and metastasis are an important regulator of metastatic potential, it can determine the location in which it will be possible to disseminate the primary tumor [18,19].

Among the factors that are active in tumor cell tropism and creating opportunities for metastatic formation in the squamous cell carcinomas of the head and neck, secretory osteopontin has been identified in the last decade as a factor without which metastasis cannot develop [20].

The idea of this research, based on a variety of communications from basic science about osteopontin and metastases, was to examine the relationship between plasma osteopontin levels and neck metastasis.

Basic work explained the influence of osteopontin in the formation of premetastatic niches, and the results of our research have established a large distribution range of osteopontin levels in patients with neck metastases. In patients with negative histopathologic findings of neck metastases, the mean value of plasma osteopontin was the lowest. These findings are compatible with the results of other studies [9,10]. Additionally, our study has shown that growing N degrees of positive neck nodes classification are characterized by growing median values and value ranges of plasma osteopontin. These results are consistent with the results of other studies [2,11,20]. However even though the OPN level was higher with a higher N degree, the highest OPN levels were in patients with histologically confirmed N2b stage. At the same time, we showed the influence of osteopontin levels in patients with HNSCC of different locations, which is not shown in other studies. These findings justify the conclusion that osteopontin plays an important role in the development of neck metastasis. 

In contrast to our results and the results of other studies that state the prognostic importance of plasma OPN level [2,11,20], the study by Lim AM et al. states the opposite [21]. According to the results of this study, high levels of OPN are not associated with adverse prognosis in HNSCC.

Nonetheless, results obtained in our study should be interpreted with caution since there was one major limitation that could be addressed in future research. An important limitation of our study was the small sample size in comparison to several other studies [21,22]. The reason for that may be found in the argumentation that this was a very sensitive group of patients, mostly older, of lower socioeconomic status who struggled to survive with malignant disease, and consequently, a low compliance for involvement in scientific research was present. In addition, the number of participants involved in our study was relatively small since our study was designed as a single-institution study conducted in a sparsely populated region. Nevertheless, the results of this research did confirm the idea that plasma osteopontin levels could be clinically relevant not only as a prognostic indicator, but could also enable more rational decisions on whether an elective neck dissection is necessary and how a selective neck dissection should be performed.

Evidently, the pathophysiological function of osteopontin in patients with malignant neck tumors is very complex and will surely be the subject of future research.

## 5. Conclusions

Osteopontin levels in the plasma of patients with negative lymph nodes are significantly lower compared to the group of patients with positive lymph nodes in the neck. Growing N degrees of positive neck lymph nodes were accompanied by growing values of OPN. Osteopontin might be important for the development of neck metastases.

## Figures and Tables

**Table 1:** Distribution of patients based on the localization of malignant head and neck tumors.

Localization of Carcinoma	Number/Total Number of Patients
Oral cavity	5/45
Epipharynx	1/45
Hypopharynx and Tonsil	1/45
Hypopharynx	4/45
Larynx	15/45
Larynx and hypopharynx	1/45
Tongue	8/45
Unknown primary site	3/45
Oropharynx	2/45
Tonsil	5/45

**Table 2:** Distribution of study participants according to the tumor–node–metastasis (TNM) classification of primary tumors.

Primary Tumor	Number/Total Number of Patients
T1	1/45
T2	13/45
T3	20/45
T4	8/45
Tx	3/45

**Table 3:** OPN values in patients with negative neck compared to all patients with positive histologic metastasis in the neck.

Nodes in the Neck-Histological Finding	*n*	OPN (ng/mL)		Hodges-Lehman Median Difference	95% CI	*p* *
		Median	25–75%			
negative	11	16.89	8.5–15.7	4.63	−2.87 to 30.95	0.03
positive	34	34.08	8.4–48.5			

Abbreviations: *: Mann Whitney U test; OPN: osteopontin.

**Table 4:** OPN levels (ng/mL) in the plasma of patients with the histologically positive metastatic neck.

Number of the Neck According to Histological Findings	*n*	OPN (ng/mL)		Test Statistic (df)	*p* *
		Median	25–75%		
N1	8	10.4	6.02–15.9	5.28 (3)	0.15
N2a	4	21.7	12.5–61.8		
N2b	14	43.9	14.6–76.3		
N2c	8	10.1	7.4–84.2		
Total	34				

Abbreviations: *: Kruskal Wallis test (Post hoc Conover); OPN: osteopontin.

**Table 5:** Plasma osteopontin levels according to the clusters.

Clusters	*n* (%)	OPN (ng/mL)		Test Statistic (df)	*p* *
		Median	25–75%		
Cluster 1: N1 neck	8 (23.5)	10.4	6.2 to 14.3	5.045 (2)	0.08
Cluster 2: N2a and N2c neck status	12 (35.3)	15.4	8.4 to 58.2		
Cluster 3: N2b	14 (41.2)	43.9	17.8 to 74.7		

Abbreviations: *: Kruskal Wallis test (Post-hoc Conover); OPN: osteopontin.

**Table 6:** Comparison of OPN plasma levels in patients based on N status of the neck.

N Status of the Neck	*n* (%)	OPN (ng/mL)		Hodges-Lehman Median Difference	95% CI	*p* *
		Median	25–75%			
**N1 vs. N2b**				33.49	2.46 to 63.7	0.02
N1 neck	8 (23.5)	10.4	6.2 to 14.3			
N2b	14 (41.2)	43.9	17.8 to 74.7			
**N1 vs. (N2a and N2c)**				6.0	−2.3 to 56.6	0.18
N1 neck	8 (23.5)	10.4	6.2 to 14.3			
N2a and N2c	12 (35.3)	15.4	8.4 to 58.2			
**N2b vs. (N2a and N2c)**				10.66	−7.83 to 38.28	0.39
N2b neck	14 (41.2)	43.9	17.8 to 74.7			
N2a and N2c	12 (35.3)	15.4	8.4 to 58.2			

Abbreviations: *: Mann Whitney U test; OPN: osteopontin.

## Data Availability

The data presented in this study are available on request from the corresponding author.
